# The GMX-Plugin for the CELLmicrocosmos MembraneEditor

**DOI:** 10.1186/1758-2946-4-S1-P49

**Published:** 2012-05-01

**Authors:** Sebastian Rubert, Christian Gamroth, André J Heissmann, Gunther Lukat, Ralf Rotzoll, Alexander Schäfer, Jens Krüger, Björn Sommer

**Affiliations:** 1 Bio-/Medical Informatics Department, Bielefeld University, Bielefeld, NRW, 33615, Germany; 2 Department of Chemistry, University of Paderborn, Paderborn, NRW, 33098, Germany

## 

Membrane research *in silico* can roughly be subdivided into three parts: the modeling, simulation and analysis process. The GMX-plugin tries to bridge the gap between these three parts represented by the tools CELLmicrocosmos MembraneEditor (CmME) and Gromacs (GMX).

CmME was developed to enable students and researchers a generation of PDB-based membranes in a fast and intuitive way without high computational requirements. From the beginning it was developed as an independent Javabased web start tool with a user-interface providing direct access to all functions implemented. The high performance of most Membrane Packing Algorithms is achieved especially by the handling of molecules as inflexible structures. The generated membranes can be exported to a PDB-file to be used with external applications [1].

One of these programs is GMX (here version 4.5.X), the well-known Molecular Dynamics package, supported and used over one decade by a very large community. It is applicable to the simulation of peptides, proteins, lipids as well as complete membranes [2].

The GMX-plugin version 1.1 is intended as an interface between CmME and GMX. It is combined with CmME on the local system and is able to access GMX on a local machine or on an external high-performance system via ssh or Unicore [3]. It is packaged with a set of lipids compatible to the Gromos 45a3 forcefield. In addition, predefined protocols exist for immediately starting a simulation of CmME-generated membranes. Custom protocols may be created, saved and reloaded by the user.

The beta version of the plugin can be downloaded at: http://Cm2.CELLmicrocosmos.org.
